# Progressive effects of single-nucleotide polymorphisms on 16 phenotypic traits based on longitudinal data

**DOI:** 10.1007/s13258-019-00902-x

**Published:** 2020-01-04

**Authors:** Donghe Li, Hahn Kang, Sanghun Lee, Sungho Won

**Affiliations:** 1 Interdisciplinary Program in Bioinformatics, Seoul National University, Seoul, Republic of Korea; 2 Biology Department, Morrissey College of Arts and Sciences, Boston College, Boston, MA USA; 3 Department of Medical Consilience, Graduate School, Dankook University, Yongin, Republic of Korea; 4 Institute of Health and Environment, Seoul National University, Seoul, Republic of Korea; 5 Department of Public Health Science, Graduate School of Public Health, Seoul National University, 1 Kwanak-ro Kwanak-gu, Seoul, 151-742 Republic of Korea

**Keywords:** Heritability, Phenotypic trait, Genomic restricted maximum likelihood, Longitudinal changes

## Abstract

**Background:**

There are many research studies have estimated the heritability of phenotypic traits, but few have considered longitudinal changes in several phenotypic traits together.

**Objective:**

To evaluate the progressive effect of single nucleotide polymorphisms (SNPs) on prominent health-related phenotypic traits by determining SNP-based heritability ($h_{snp}^{2}$) using longitudinal data.

**Methods:**

Sixteen phenotypic traits associated with major health indices were observed biennially for 6843 individuals with 10-year follow-up in a Korean community-based cohort. Average SNP heritability and longitudinal changes in the total period were estimated using a two-stage model. Average and periodic differences for each subject were considered responses to estimate SNP heritability. Furthermore, a genome-wide association study (GWAS) was performed for significant SNPs.

**Results:**

Each SNP heritability for the phenotypic mean of all sixteen traits through 6 periods (baseline and five follow-ups) were significant. Gradually, the forced vital capacity in one second (FEV1) reflected the only significant SNP heritability among longitudinal changes at a false discovery rate (FDR)-adjusted 0.05 significance level ($h_{snp}^{2} = 0.171$, FDR = 0.0012). On estimating chromosomal heritability, chromosome 2 displayed the highest heritability upon periodic changes in FEV1. SNPs including rs2272402 and rs7209788 displayed a genome-wide significant association with longitudinal changes in FEV1 (*P *= 1.22 × 10^−8^ for rs2272402 and *P *= 3.36 × 10^−7^ for rs7209788). De novo variants including rs4922117 (near *LPL*, *P *= 2.13 × 10^−15^) of log-transformed high-density lipoprotein (HDL) ratios and rs2335418 (near *HMGCR*, *P *= 3.2 $\times$ 10^−9^) of low-density lipoprotein were detected on GWAS.

**Conclusion:**

Significant genetic effects on longitudinal changes in FEV1 among the middle-aged general population and chromosome 2 account for most of the genetic variance.

**Electronic supplementary material:**

The online version of this article (10.1007/s13258-019-00902-x) contains supplementary material, which is available to authorized users.

## Introduction

Single nucleotide polymorphism (SNP)-based heritability ($h_{snp}^{2}$) indicates the relative proportion of genetic variance explained based on SNPs used for genome-wide association studies (GWASs). For $h_{snp}^{2}$ estimation, the genomic restricted maximum likelihood (GREML) for linear mixed models (LMMs) is often implemented in the genetic complex trait analysis (GCTA) tool (Yang et al. 2011). GREML first calculates the genetic relatedness matrices (GRM), which are used as variance–covariance matrices for random effects. The significance of estimates obtained through GREML depends on the study design; if it is applied to family-based samples, it displays pedigree-based heritability, but for unrelated subjects, it estimates $h_{snp}^{2}$ (Kim et al. 2015; Yang et al. 2017). Estimation of $h_{snp}^{2}$ involves considerable differences across not only methodologies but also procedures requiring careful interpretation of results (Evans et al. 2018; Ni et al. 2018). Moreover, the estimated heritability is potentially biased and misleading owing to measurement errors at various degrees. To overcome these challenges, the heritability determined from longitudinal data is more reliable than that determined from cross-sectional data. While most studies on $h_{snp}^{2}$ focused on the primary effect of SNPs, significant effects of SNPs on the average annual differences indicate the SNP-by-age interaction. Numerous examples illustrate the importance of age on longitudinal changes (Genetic epidemiologic studies on age-specified traits. NIA Aging and Genetic Epidemiology Working Group 2000; Nishimura et al. 2012; van de Pol and Verhulst 2006). For instance, one study showed that an annual decline in lung function is associated with age (Kim et al. 2016), and another study reported a genetic influence on changes in both lipoprotein risk factors and systolic blood pressure over a decade (Friedlander et al. 1997). Therefore, the $h_{snp}^{2}$ should be estimated on the basis of not only the mean of observed traits but also changes in the sufficient period. Hence, we applied a two-stage approach, which is a convenient method of analyzing longitudinal data by combining linear regression models to investigate the effect of SNPs on both average and longitudinal differences in phenotypic traits.

In this study, we investigated the magnitude of SNPs effect on average and longitudinal differences by using both genomic data and 16 phenotypic traits associated with major health indices using a phenotype-genotype dataset of unrelated individuals in a community-based cohort and evaluated their importance. Except for baseline, each phenotype was objectively measured every 2 years for 10-year follow-up, and six repeated measurements (maximum) were obtained for each individual. For each subject, both the average phenotypic traits and their longitudinal changes were estimated via subject-specific regression analysis, using intercepts and coefficients of ages, respectively. Each $h_{snp}^{2}$ value was estimated using GCTA. Our results show that lung function has the only significant $h_{snp}^{2}$ for longitudinal changes, while all average phenotypes of the 16 traits yielded a significant $h_{snp}^{2}$ value. Furthermore, the GWAS revealed certain novel genome-wide significant SNPs associated with the phenotypes analyzed herein.

## Materials and Methods

### Ethics approval and consent to participate

The respective Institutional Review Board (IRB) of Seoul National University reviewed and approved the informed consent, the study protocols and other documents (Permit No. E1605/002-003). All methods were performed in accordance with the relevant guidelines and regulations.

### Korea Associated Resource (KARE) cohort data

Korea Associated Resource (KARE) data are based on a community-based epidemiological study and comprises subjects residing in Ansan (urban area) and Ansung (rural area) in the Gyeonggi Province of South Korea (Cho et al. 2009). A baseline survey was completed in 2001–2002, and 10,030 participants aged 40–69 years were recruited. After that, biennial repeated surveys were conducted, and the last survey were completed in 2013–2014 (Kim et al. 2017). Six different surveys were conducted in total. These measurement periods are indicated as periods 1–6 throughout this study, each with a different number of subjects (e.g., period 1: 8543 subjects [4052 male, 4491 female]; period 6: 5391 subjects [2502 male, 2889 female]). The number of overlapping subjects throughout the six periods was 4306 (2009 male, 2297 female). Among these, subjects whose traits were measured at least three times were considered, and 6843 participants (3273 male, 3570 female) were assessed in total.

Many participant phenotypes were recorded by trained interviewers through questionnaires and clinical measurement, and we only considered the 16 quantitative traits that they were measured objectively and associated with major health indices; these were classified into four groups: anthropometric, biochemistry, cardiopulmonary, and red blood cell traits (Table 1). As glycated hemoglobin (HbA1c), fasting blood glucose (GLU0), high-density lipoprotein (HDL), triglycerides (TG), and systolic blood pressure (SBP) displayed skewed distributions, they were log-transformed and denoted by log(HbA1c), log(GLU0), log(HDL), log(TG), and log(SBP), respectively. The missing rate of HbA1c was larger than 0.5 at period 2 and was excluded from further analyses. For each trait, subjects with more than three measurement observations were assessed.

**Table 1:** Sixteen phenotypic traits associated with major health indices

Anthropomorphic traits	Height, Waist, Weight, body-mass index (BMI)
Biochemistry traits	
Glucose	Glycated hemoglobin (HbA1c), Fasting blood glucose (GLU0)
Cholesterol	Low-density lipoprotein (LDL), high-density lipoprotein (HDL), total cholesterol (TCHL), triglyceride (TG)
Cardiopulmonary traits	
Blood pressure	Systolic blood pressure (SBP), diastolic blood pressure (DBP)
Lung capacity	Predicted forced vital capacity (FVC) %, predicted forced expiratory volume in one second (FEV1) %, predicted FEV1/FVC %
Red blood cell traits	Hemoglobin levels (Hb)

### Genotypes

**Fig. 1:**
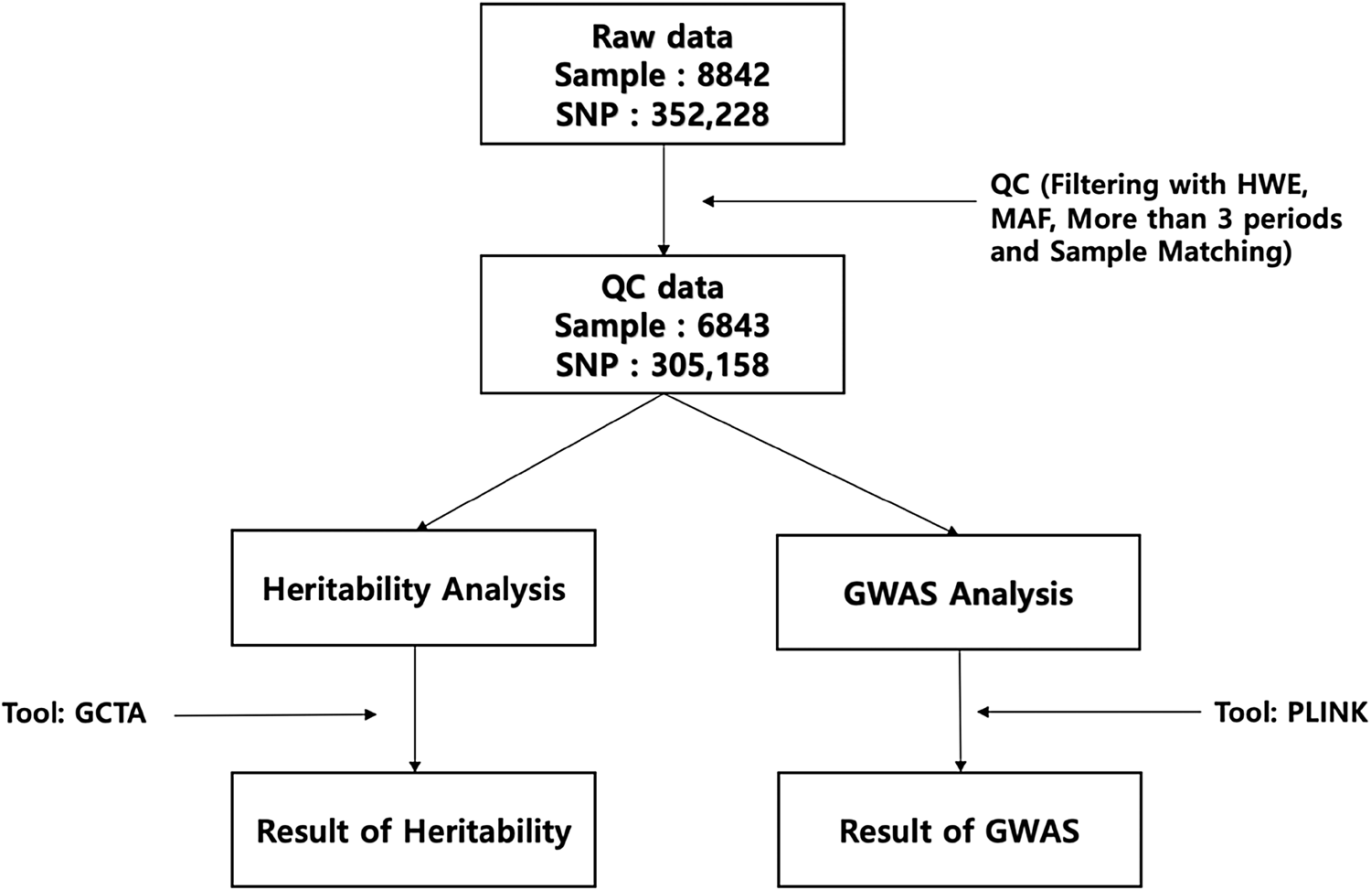
A schematic representation of heritability analysis and the genome-wide association study

Genotype data for the KARE cohort were obtained using the Affymetrix Genome-Wide Human SNP array 5.0 (Cho et al. 2009). Quality control (QC) analysis of SNPs and subjects was conducted using PLINK (Purcell et al. 2007) and ONETOOL (Song et al. 2018). SNPs with *P*-values from Hardy–Weinberg equilibrium (HWE) analysis < 10^−5^, minor allele frequencies (MAFs) < 0.05, and genotype call rates < 95% were excluded. Furthermore, subjects with missing genotype call rates > 5% or sex-based inconsistencies were excluded. The missing genotypes for typed SNPs were imputed on the basis of the 1000-genome sequence reference data. After QC analysis, 305,158 SNPs were analyzed for SNP heritability and GWAS (Fig. 1).

### Data availability

The data used in this study underwent an application process according to the Korean Genome and Epidemiology study data access policy, and can be downloaded from following website: http://nih.go.kr/index.es?sid=a5#a.

### Determination of phenotypic averages and longitudinal changes in each subject

The phenotypic averages and longitudinal changes for each subject were determined and used to estimate SNP heritability and for the GWAS. Significant differences in phenotypic variances were observed for each period, and such heteroscedasticity was considered for phenotypic averages and longitudinal changes for each subject as follows. First, the linear regression model for subjects of the same period was adjusted for traits. The effect of sex, age, and top 10 principal component (PC) scores estimated from the genetic relationship matrix were adjusted as covariates. Considering $w_{jk}$ as the residual variances of trait *k* (*k *= 1, …, 16) at the period *j*, the following linear regression model was adjusted with repeated measures for each subject *i* as follows:

1 $$y_{ijk} = \beta_{ik0} + \beta_{ik1} \left( {age_{ij} - \overline{{age_{i} }} } \right) + \varepsilon_{ijk} , \varepsilon_{ijk} \sim N\left( {0,\frac{1}{{w_{jk} }}\sigma_{ik}^{2} } \right)$$

Here, *i* indicates the *i*th subject, and $\overline{{age_{i} }}$ indicates the mean of ages at the observed time points. In the regression model (1), $y_{ijk}$ is the *j*th period observed value of subject *i* for trait *k*, $\beta_{ik0}$ indicates the expected phenotypic mean of subject *i* for trait *k* when he or she is $\overline{{age_{i} }}$ years old, and $\beta_{ik1}$ is the average longitudinal change in trait *k*. The estimated values of $\beta_{ik0}$ and $\beta_{ik1}$ were used to estimate the heritability and for GWAS analysis. For convenience, both are denoted by $B_{0}$ and $B_{1}$, respectively.

### Estimation of heritability

$B_{0}$ and $B_{1}$ were separately used to estimate SNP heritability. Analyses were conducted using GCTA (Yang et al. 2011), and the restricted maximum likelihood estimator was used. Effects of age and sex were adjusted as covariates.

### GWAS analysis

$B_{0}$ and $B_{1}$ were separately analyzed to identify disease susceptibility loci for 16 different traits. The $\overline{{age_{i} }}$, sex, and 10 PC scores estimated from the genetic relationship matrix were included as covariates to adjust the population stratification.

## Results

### Estimation of heritability

A schematic representation of the heritability analysis and genome-wide association study is shown in Fig. 1. For 16 different traits of 6843 subjects, the mean and standard deviation values of each trait at period 1 are shown in Table 2 (see Table 1 for detailed information). Some missing values resulted in differences in the total number of subjects depending on the phenotype, and the sample sizes of those traits and descriptive statistics including sex and age were summarized.

**Table 2:** Descriptive statistics of 16 traits

Trait	Trait (baseline)		Total (N)	Female		Age	
	Mean	SD		N	%	Mean	SD
Height (cm)	160.11	8.63	6823	3557	52.13%	51.90	8.69
Waist (cm)	82.63	8.70	6835	3567	52.19%	51.90	8.69
Weight (kg)	63.24	10.10	6822	3556	52.13%	51.90	8.69
BMI (kg/m^2^)	24.62	3.10	6822	3556	52.13%	51.90	8.69
HbA1c (%)	5.74	0.82	6329	3321	52.47%	51.87	8.62
GLU0 (mg/dl)	86.73	19.41	6728	3514	52.23%	51.85	8.67
TG (mg/dl)	161.47	103.19	6840	3568	52.16%	51.91	8.70
LDL (mg/dl)	115.00	32.89	6840	3568	52.16%	51.91	8.70
HDL (mg/dl)	44.69	9.91	6840	3568	52.16%	51.91	8.70
TCHL (mg/dl)	191.92	35.09	6840	3568	52.16%	51.91	8.70
SBP (mmHg)	121.12	18.10	6843	3570	52.17%	51.91	8.70
DBP (mmHg)	80.19	11.33	6843	3570	52.17%	51.91	8.70
Hb (g/dl)	13.61	1.57	6840	3568	52.16%	51.91	8.70
FVC (%predicted)	104.76	14.17	4291	2135	49.76%	50.37	8.17
FEV1 (%predicted)	112.27	16.62	4290	2134	49.74%	50.37	8.16
FEV1/FVC (predicted)	74.89	1.77	4291	2135	49.76%	50.37	8.17

**Fig. 2:**
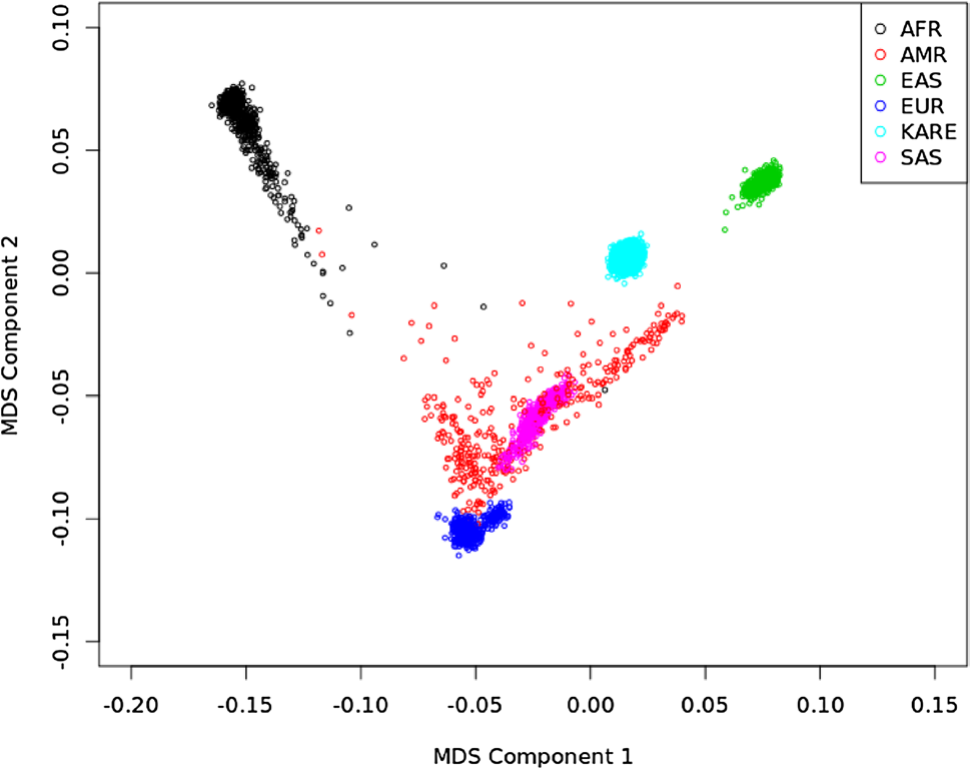
Population structures identified via a multidimensional scaling (MDS) plot. This plot shows that our analyses (KARE) are not affected by population stratification. AFR, AMR, EAS, EUR, and SAS indicate African, Ad Mixed American, East Asian, European, and South Asian populations, respectively, from the 1000 Genomes Project

For those 6843 subjects, a multidimensional scaling (MDS) plot was generated (Fig. 2). As shown in Fig. 2, subjects from the 1000 Genomes Project were also included, and our analyses were not affected by population stratification.

**Fig. 3:**
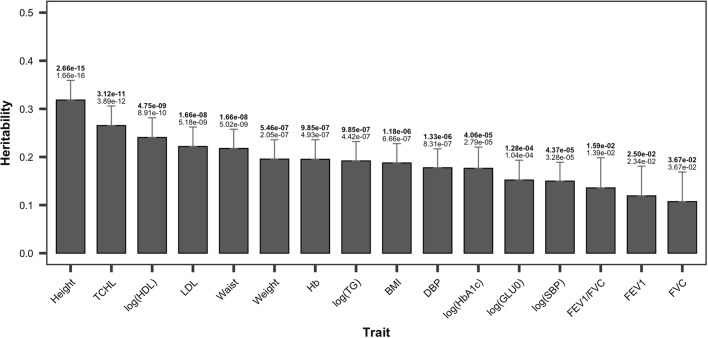
Single-nucleotide polymorphism heritability estimates of 16 traits with $B_{0}$ as the response. Error bars correspond to standard error values. The values above the error bar are *P* values and false discovery rate (FDR; bold)

We calculated the descriptive statistics for $B_{0}$ and $B_{1}$ (Table 3, see “Materials and methods” for details). $B_{0}$ in Eq. (1) indicates the means of the predicted traits at $\overline{{age_{i} }}$ years of age. $B_{1}$ stands for the longitudinal changes in the traits of each subject. Table 3 shows that the means of $B_{0}$ are similar to those of period 1. Means of $B_{1}$ were generally closer to 0. Figure 3 shows the estimates of heritability with $B_{0}$ as the response in the GREML model, and the estimated heritability of height for the data peaked at 0.318 (*P *= 1.665 $\times$ 10^−16^, FDR = 2.664 $\times$ 10^−15^). The subsequent three highest heritability traits were total cholesterol (TCHL), log(HDL), and low-density lipoprotein (LDL), with values of 0.265 (*P *= 3.895 $\times$ 10^−12^, FDR = 3.116 $\times$ 10^−11^), 0.241 (*P *= 8.911 $\times$ 10^−10^, FDR = 4.753 $\times$ 10^−9^), and 0.222 (*P *= 5.178 $\times$ 10^−9^, FDR = 1.657 $\times$ 10^−8^), respectively. These three traits are cholesterol-related. The heritability of waist was 0.218 (*P *= 5.016 $\times$ 10^−9^, FDR = 1.657 $\times$ 10^−8^) and that of weight was 0.196 (*P *= 2.046 $\times$ 10^−7^, FDR = 5.456 $\times$ 10^−7^). The heritability was 0.195 for Hb (*P *= 4.926 $\times$ 10^−7^, FDR = 9.852 $\times$ 10^−7^) and 0.192 for log(TG) (*P *= 4.419 $\times$ 10^−7^, FDR = 9.852 $\times$ 10^−7^). The heritability of the other traits with an FDR larger than 1 $\times$ 10^−6^ were less than 0.19.

**Table 3:** Summary of $B_{0}$ and $B_{1}$ of 16 traits

Trait	$B_{0}$				$B_{1}$			
	Mean	SD	Min	Max	Mean	SD	Min	Max
Height	159.906	8.724	130.241	187.866	− 0.060	0.139	− 2.168	0.747
Waist	83.743	8.480	58.333	121.591	0.184	0.692	− 4.968	6.904
Weight	62.860	9.931	30.532	105.355	− 0.094	0.480	− 3.739	2.657
BMI	24.531	2.992	14.197	38.831	− 0.019	0.185	− 1.486	1.048
log(HbA1c)	1.737	0.107	1.256	2.441	0.002	0.011	− 0.093	0.157
log(GLU0)	4.538	0.149	4.260	5.733	0.012	0.018	− 0.166	0.171
log(TG)	4.834	0.423	3.584	7.189	− 0.012	0.057	− 0.409	0.412
LDL	120.111	25.757	11.833	281.590	0.193	4.065	− 29.218	28.549
log(HDL)	3.782	0.193	3.100	4.567	− 0.001	0.024	− 0.211	0.135
TCHL	194.208	28.114	97.986	343.106	− 0.120	4.468	− 34.599	29.468
log(SBP)	4.768	0.114	4.461	5.156	− 0.001	0.017	− 0.122	0.086
DBP	78.252	8.239	50.639	111.556	− 0.259	1.358	− 12.330	8.066
Hb	13.695	1.370	7.764	18.889	0.022	0.147	− 1.641	1.468
FVC	104.541	13.466	46.629	162.844	− 0.090	2.478	− 13.065	13.685
FEV1	111.128	16.295	38.951	184.532	− 0.239	2.575	− 16.022	15.620
FEV1/FVC	73.945	1.809	67.654	78.000	− 0.213	0.127	− 1.246	1.244

**Fig. 4:**
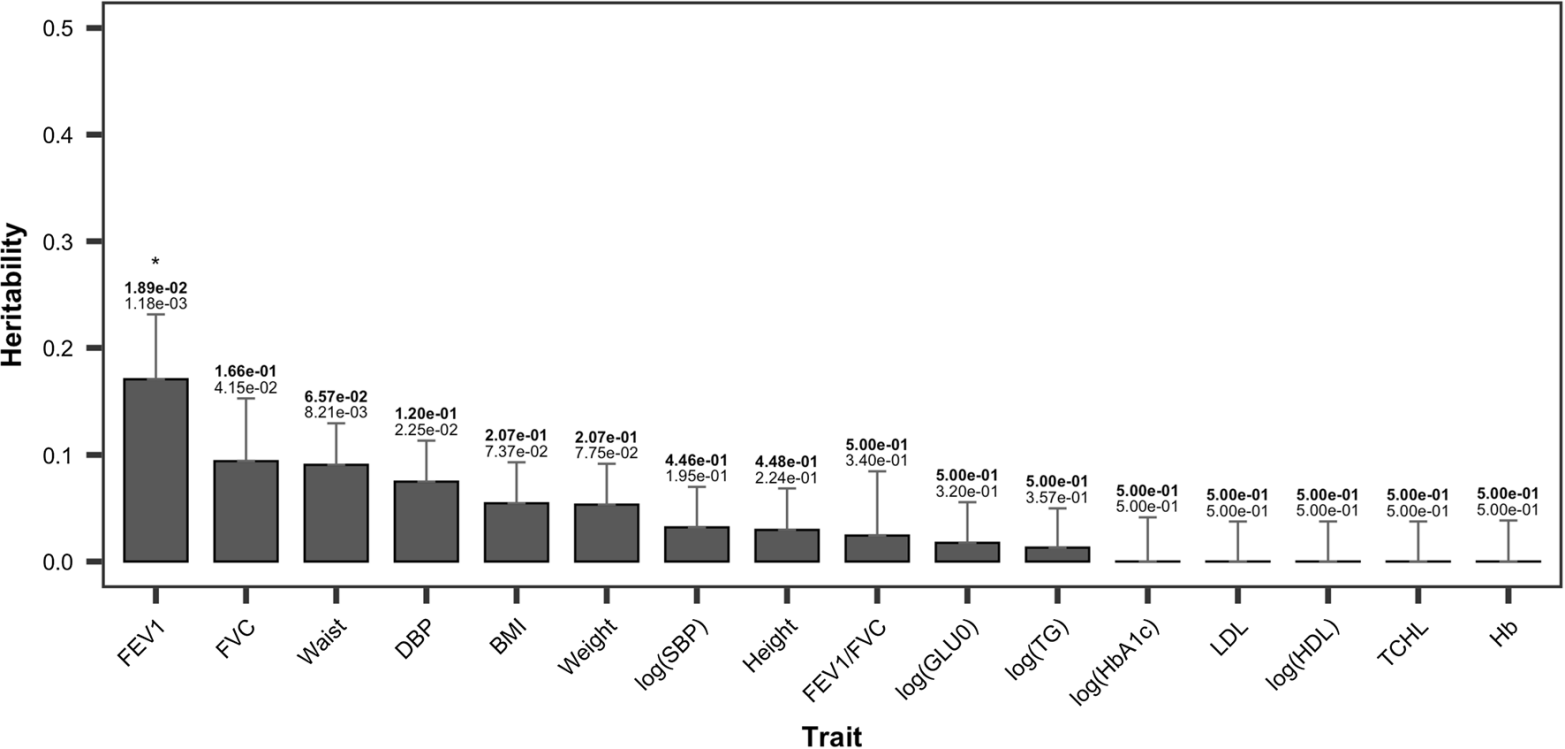
Single-nucleotide polymorphism heritability estimates of 16 traits with $B_{1}$ as the response. Error bars correspond to standard error values. The values above the error bar are *P* values and the false discovery rate (FDR; bold), and “*” indicates significant findings at an FDR of 0.05

Figure 4 shows the heritability estimates for $B_{1}$, which are generally less than those for $B_{0}$, and we found that lung function traits FVC and FEV1, wasit, diastolic blood pressure (DBP), BMI, and log(SBP) are relatively high. The highest heritability estimate was observed for FEV1 (0.171) and its FDR-adjusted *P* value was 0.0189. The heritability estimates of other traits were less than 0.1. The second highest heritability was 0.0941 for FVC, and its FDR-adjusted *P* value was 0.166. The heritability of waist was also relatively higher than that of other traits. Waist heritability and the FDR-adjusted *P* values were 0.0082 and 0.0657, respectively. The higher heritability estimates for $B_{1}$ indicate that the decreasing/increasing rates were associated with genetic factors. Height displayed the highest heritability estimates for $B_{0}$, but its estimate for $B_{1}$ was low (0.0297). Height does not usually change after the age of 20, and its lower value here is probably attributable to it. For the other traits including log(HbA1c), LDL, log(HDL), TCHL, and Hb levels, SNP heritability estimates tended towards 0.

**Fig. 5:**
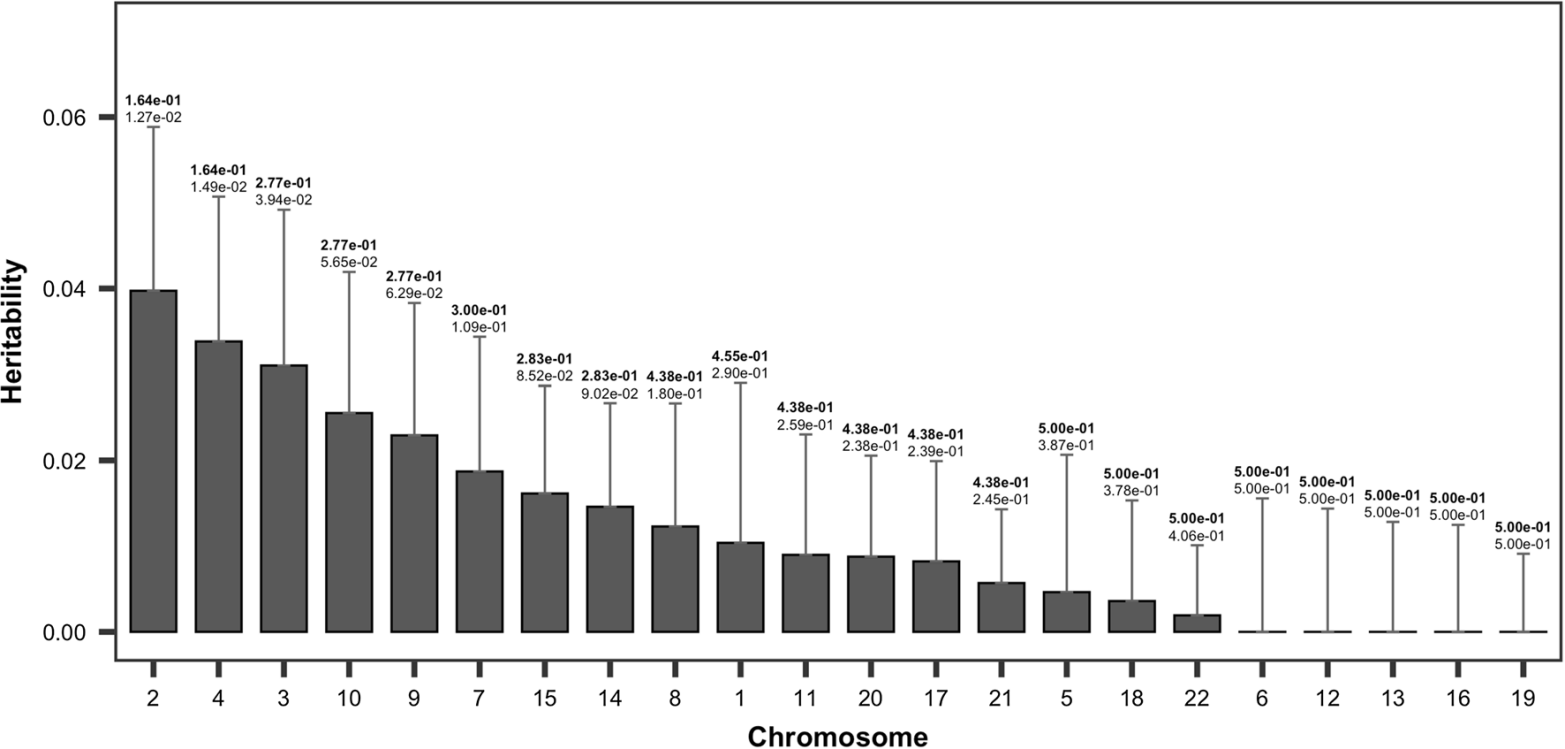
Single-nucleotide polymorphism heritability estimates of FEV1 based on chromosomes with $B_{1}$ as the response. Error bars correspond to standard error values. The values above the error bar are *P* values and false discovery rate (FDR; bold)

**Fig. 6:**
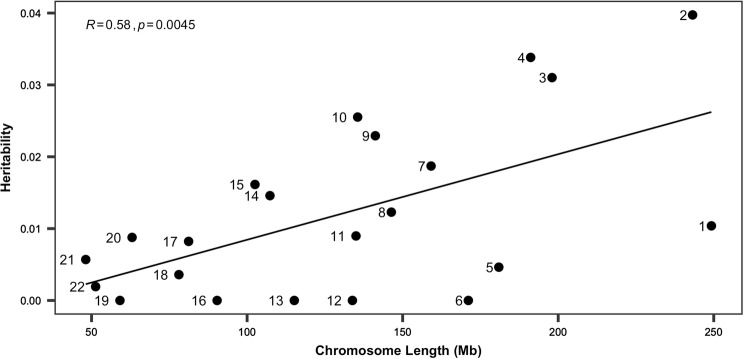
Correlation between chromosome length and chromosome-specific heritability. Numbers near dots are the chromosome numbers. Black line is the estimated regression line

Furthermore, we determined chromosomal heritability estimates of FEV1, which displayed the highest heritability in the $B_{1}$ model. Consequently, chromosome 2 accounted for the highest proportion of phenotypic variance ($h_{snp}^{2}$ = 0.0397), albeit with a high standard error (Fig. 5).There was a significant positive correlation between chromosome length and heritability (*r *= 0.58, *P *= 0.0045) in FEV1 (Fig. 6).

### Genome-wide association studies

$B_{0}$ and $B_{1}$ were considered responses for GWAS. Tables 4 and 5 show genome-wide significant SNPs at a significance level of 1 × 10^−7^. Subsequent findings are summarized in the Supplementary material (Tables S1 and S2). Table 4 shows that SNPs have relatively lower *P* values for log(TG) and log(HDL) than any other trait. The most significant variant for log(TG) was rs6589566 in *ZPR1* with a *P*-value of 7.9 $\times$ 10^−38^, the lowest *P* value among all 16 traits. Furthermore, *ZPR1* is associated with TG (Coram et al. 2013). The most significant variant of log(HDL) was rs16940212 with a *P* value of 2.08 $\times$ 10^−18^ in *ALDH1A2*, which is associated with HDL (Spracklen et al. 2017). Certain other significant variants were significantly associated with proximal genes and with traits assessed herein. The variant rs180349 (*P *= 8.86 $\times$ 10^−35^) of log(TG) was proximal to *BUD13*, which is associated with TG (Hoffmann et al. 2018). The variant rs17482753 (*P *= 3.199 $\times$ 10^−18^) is proximal to *LPL*, which was strongly associated with HDL (Hoffmann et al. 2018). Herein, we also detected some de novo variants including rs4922117 (*P *= 2.13 $\times$ 10^−15^) of log(HDL) and rs2335418 (*P *= 3.2 $\times$ 10^−9^) of LDL, which were previously unknown; however, both their proximal genes *LPL* and *HMGCR* are significantly associated with each trait (Hoffmann et al. 2018). The Manhattan Plot and QQ plot for the model with $B_{0}$ as the response are provided in the Supplementary material (Figures S1 and S2).

**Table 4:** Results of the genome-wide association study with $B_{0}$ as the response

Trait	SNP	CHR	BP1	BP2	A1	A2	GP	GENE	MAF	HWE_P	BETA	*P*
log(GLU0)	rs1799884	7	44,229,068	44,229,068	A	G	Upstream	GCK	0.1872	0.9051	0.01889	5.62E−09
log(GLU0)	rs7754840	6	20,661,250	20,661,250	C	G	Intronic	CDKAL1	0.478	1	0.01488	6.25E−09
Hb	rs5756505	22	37,467,354	37,467,354	C	G	Intronic	TMPRSS6	0.4979	0.9229	0.1125	2.61E−13
Hb	rs3768751	2	46,346,716	46,346,716	G	A	Intronic	PRKCE	0.1796	1	− 0.113	1.79E−08
log(HbA1c)	rs7754840	6	20,661,250	20,661,250	C	G	Intronic	CDKAL1	0.478	1	0.01254	5.41E−11
log(HDL)	rs16940212	15	58,694,020	58,694,020	T	G	Intergenic	ALDH1A2	0.3414	0.9786	0.02988	2.08E−18
log(HDL)	rs17482753	8	19,832,646	19,832,646	T	G	Intergenic	LPL(dist = 7876),SLC18A1(dist = 169,720)	0.1241	0.1333	0.04232	3.20E−18
log(HDL)	rs4922117	8	19,852,586	19,852,586	G	A	Intergenic	LPL(dist = 27,816),SLC18A1(dist = 149,780)	0.2077	0.418	0.0316	2.13E−15
LDL	rs599839	1	109,822,166	109,822,166	G	A	Downstream	PSRC1	0.06456	0.1925	− 5.886	1.53E−11
LDL	rs12654264	5	74,648,603	74,648,603	T	A	Intronic	HMGCR	0.4758	0.8085	− 2.625	1.41E−09
LDL	rs2335418	5	74,603,479	74,603,479	G	A	Intergenic	ANKRD31(dist = 70,776),HMGCR(dist = 29,514)	0.4232	0.5689	− 2.6	3.20E−09
LDL	rs4045166	5	74,909,446	74,909,446	G	C	Intronic	ANKDD1B	0.3326	0.3137	2.727	3.55E−09
LDL	rs10942739	5	74,786,083	74,786,083	T	C	Intronic	COL4A3BP	0.3325	0.276	2.709	4.61E−09
LDL	rs688	19	11,227,602	11,227,602	T	C	Exonic	LDLR	0.136	0.797	3.402	6.63E−08
TCHL	rs599839	1	109,822,166	109,822,166	G	A	Downstream	PSRC1	0.06456	0.1925	− 6.822	1.19E−12
TCHL	rs780092	2	27,743,154	27,743,154	G	A	Intronic	GCKR	0.3248	0.2948	− 3.365	4.63E−11
TCHL	rs17321515	8	126,486,409	126,486,409	T	C	Intergenic	TRIB1(dist = 35,762),LINC00861(dist = 448,358)	0.4425	0.04178	2.782	4.20E−09
TCHL	rs1881396	2	27,844,601	27,844,601	G	T	UTR3	ZNF512	0.3313	0.8699	− 2.793	3.18E−08
TCHL	rs6861279	5	74,919,409	74,919,409	T	C	Intronic	ANKDD1B	0.3386	0.1772	2.784	3.99E−08
TCHL	rs6734059	2	27,808,154	27,808,154	C	T	Intronic	ZNF512	0.3357	0.8921	− 2.724	6.36E−08
log(TG)	rs6589566	11	116,652,423	116,652,423	C	T	Intronic	ZPR1	0.2169	0.3545	0.1113	7.90E−38
log(TG)	rs180349	11	116,611,827	116,611,827	A	T	Intergenic	LINC00900(dist = 980,909),BUD13(dist = 7059)	0.2265	0.752	0.1065	8.86E−35
log(TG)	rs10503669	8	19,847,690	19,847,690	T	G	Intergenic	LPL(dist = 22,920),SLC18A1(dist = 154,676)	0.1207	0.003444	− 0.08902	1.63E−16
log(TG)	rs780094	2	27,741,237	27,741,237	C	T	Intronic	GCKR	0.4626	0.9806	− 0.05656	2.60E−15
log(TG)	rs7115242	11	116,908,283	116,908,283	T	C	Intronic	SIK3	0.2796	0.133	0.05987	8.66E−14

Only the significant variants with *P* values less than 1 × 10^−7^ in each trait are included. The more significant results are listed in the supplement material (Table S1)

**Table 5:** Results of the genome-wide association study with $B_{1}$ as the response

Trait	SNP	CHR	BP1	BP2	A1	A2	GP	GENE	MAF	HWE_P	BETA	*P*
FEV1	rs2272402	3	11,075,461	11,075,461	A	G	Intronic	SLC6A1	0.07363	0.1473	− 0.5823	1.22E−08
FVC	rs2272402	3	11,075,461	11,075,461	A	G	Intronic	SLC6A1	0.07363	0.1473	− 0.595	1.40E−09

Only the variants with *P* values less than 1 × 10^−7^ are included. The more variants under suggestive threshold (*P* values less than 1 × 10^−5^) are listed in the supplement material (Table S2)

Table 5 shows the results of GWAS for $B_{1}$. Based on the results, rs2272402 (*SLC6A1*) was the most significant variant both in FEV1 (*P *= 1.22 $\times$ 10^−8^) and FVC (*P *= 1.40 $\times$ 10^−9^) (Regional plots are shown in Supplementary Figures S5 and S7), and the *SLC6A1* enhancer was associated with lung function. Other variants including rs7209788 (*NARF*, *P *= 3.36 $\times$ 10^−7^) for FEV1 and rs2668162 (*FAM19A1*, *P *= 6.18 $\times$ 10^−7^) for FVC had *P*-values less than the 1 $\times$ 10^−6^ threshold (S2 Table). From the regional plot (Supplementary Figure S6), we found that rs4789777(*HEXDC*, *P *= 4.599 $\times$ 10^−6^) is highly correlated with rs7209788 of FEV1. The Manhattan Plot and QQ plot for the model with $B_{1}$ as the response are provided in the Supplementary material (Figures S3 and S4).

## Discussion

In this study, SNP-based heritability estimates of 16 phenotypic traits were estimated longitudinal data from a 10-year follow-up of the KARE cohort. The GCTA tool was used with a two-stage approach to determine the heritability estimate of phenotypic mean and longitudinal changes in each trait. Moreover, chromosomal heritability estimates were determined and GWAS analyses were performed using the same approach. Overall, heritability estimates within the population-based cohort including KARE are potentially lower than those of pedigree or twin studies for all 16 traits, regardless of whether the response is $B_{0}$ that phenotypic mean of traits or $B_{1}$ which stands for the changes by time of traits. For example, the heritability of height herein was estimated to be approximately 0.318 with $B_{0}$ as the response, which is lower than the conventional heritability estimate of height of approximately 0.8 based on the assumption-free model (Visscher et al. 2006). In the case of TCHL and LDL, each heritability estimate was determined to be 0.265 and 0.22, respectively, which are also lower than the heritability estimates of 0.67 and 0.69 for TCHL and LDL, respectively, on familial and pedigree analysis (van Dongen et al. 2013). The underlying reason may be explained on the basis of the missing heritability, which describes the difference in values between heritability estimated via GWAS and via familial studies (Sandoval-Motta et al. 2017). However, systemic inflation of estimated heritability estimates of polygenic phenotypes in familial studies may be confounded owing to a shared environment or environment-dependent genetic effects (Robinson et al. 2017). Therefore, the population-based design similar to that of the present study potentially represents the average genetic effects regardless of various confounding environmental factors.

Based on the present $B_{0}$ and $B_{1}$ model, the heritabilities of $B_{1}$ are markedly lower than those of $B_{0}$, indicating that most of the genetic variance of traits are not temporally influenced. Here, $B_{0}$ was not determined from the baseline measurements of traits but rather the average values of repeated measurements to yield a more robust and reasonable result. If baseline measurement and longitudinal changes $(B_{1} )$ calculated from those were considered responses during the estimation of heritability, the estimate may have been potentially inaccurate owing to the correlation between baseline and $B_{1}$ values. Thus, by applying a regression model to estimate the average $B_{0}$ and longitudinal changes $B_{1}$, the effect of $B_{0}$ on $B_{1}$ in each subject could be removed.

On GWAS, the two-stage model elucidated significant variants associated with the traits and their changes in the longitudinal data. We confirmed several proven variants and identified some other significant unreported variants. In the case of the $B_{0}$ model, rs4922117 (*P *= 2.13 $\times$ 10^−15^) of log (HDL) and rs2335418 (*P *= 3.2 $\times$ 10^−9^) of LDL were both unreported; however, their proximal genes *LPL* and *HMGCR*, respectively, were significantly associated with each trait (Hoffmann et al. 2018). Furthermore, unreported genes, such as rs180349, including non-coding SNPs with a significant *P* value for TG are proximal to *BUD13*, which is strongly associated with TG (Hoffmann et al. 2018). Variants including rs17482753 also had significant *P* values and was proximal to *LPL*, which is strongly associated with the HDL trait (Hoffmann et al. 2018). In the $B_{1}$ model, rs2272402 (*SLC6A1*, *P *= 1.22 $\times$ 10^−8^) was significant in both FEV1 and FVC lung function. The *SLC6A1* enhancer is associated with pulmonary function. Therefore, the present results are concurrent with previous findings regarding genes associated with each phenotype.

Among the 16 phenotypic traits in this study, only FEV1 displayed longitudinally significant heritability herein (Fig. 4), thus reliably reflecting the physiological state of the lungs and airways and acting as a predictor of morbidity and mortality in the general population; FEV1 is also widely used to define chronic obstructive pulmonary disease (COPD) (Young et al. 2007). Lung function develops in early life, peaks at a specific time point in early adulthood, and subsequently declines with age. Therefore, the decline of lung function in middle-aged and older individuals is suggested to be heritable in the general population (Gottlieb et al. 2001). However, longitudinal studies on FEV1 and FEV1/FVC have suggested several significant genetic regions that markedly differ from the numerous genetic variants associated with lung function, with FEV1 being estimated at a single time point (John et al. 2017; Tang et al. 2014). Hence, gene-environment interactions and significant genetic heterogeneity in lung function have been  observed in diseases such as asthma or COPD (Hansel et al. 2013; Imboden et al. 2012). Accordingly, the present study included the middle-aged general population with similar environmental exposure without specific lung diseases, thus suggesting that intact FEV1 decreased due to aging. Therefore, the present results show that FEV1 has significant SNP heritability for longitudinal changes (FDR = 0.0012 for FEV1).

This study has several limitations. First, the analysis of new variants in the present GWAS was not replicated for other cohorts. Second, the two-stage approach is statistically inefficient even though it is computationally fast. However, the sample size was very large, which hopefully minimized this problem. Furthermore, we considered subjects with at least three or more measurements, which potentially minimize statistical power loss. Third, gene-environment interactions were not analyzed, although the estimation of random effects in the mixed model was elusive. Fourth, GCTA itself has limitations for reasons such as data overfitting and skewed singular values (Kumar et al. 2016). Though our study optimized parameters to attain accurate results using GCTA, our sample size might have resulted in certain variations in comparison with other large studies. Furthermore, the issue regarding missing heritability was inevitable to an extent because the Affymetrix genotypic array represents only common variants for SNPs, while rare genetic SNP variants were not included herein (Bandyopadhyay et al. 2017).

Despite the aforementioned limitations, our study elucidates heritability estimates via a two-stage approach using a mixed model in GCTA and a GWAS, which further determines longitudinal change effects independently with a linear model, followed by estimating heritability using regression coefficients. This approach provides a reasonable and easy method to estimate heritability in longitudinal data and potentially assess both heritability of the phenotypic mean and changes through several periods. Essentially, our results show that significant SNP heritability is objectively confirmed for longitudinal changes in lung function decline including FEV1 in comparison with other health-related indices. Therefore, genetic studies on longitudinal FEV1 decline among the middle-aged general population and chromosome 2, which attributes the most in genetic variance should be encouraged.

## Electronic supplementary material

Below is the link to the electronic supplementary material.

Supplementary material 1 (DOCX 2019 kb)
