# Supplementary material for: Progressive effects of single-nucleotide polymorphisms on 16 phenotypic traits based on longitudinal data
Source: Genes Genomics. 2020 Jan 4;42(4):393–403. doi: 10.1007/s13258-019-00902-x (PMC7113194; doi:10.1007/s13258-019-00902-x)
Supplement: Supplementary file 1 — Supplementary material 1 (DOCX 2019 kb) [file 13258_2019_902_MOESM1_ESM.docx]

**Progressive effect of single-nucleotide polymorphisms on 16 Phenotypic Traits with Longitudinal Data**

Donghe Li^1^, Hahn Kang^2^, Sanghun Lee^3^*, Sungho Won^1,4,5^*

^1^ Interdisciplinary Program in Bioinformatics, Seoul National University, Seoul, Republic of Korea

^2^ Biology Department, Morrissey College of Arts and Sciences, Boston College, MA, United States.

^3^ Department of Medical Consilience, Graduate School, Dankook University, Yongin, Republic of Korea

^4^ Institute of Health and Environment, Seoul National University, Seoul, Republic of Korea.

^5^ Department of Public Health Science, Graduate School of Public Health, Seoul National University, Seoul, Republic of Korea

Table S1. Result of GWAS with $\boldsymbol{B}_{\mathbf{0}}$as response.

| **Trait** | **SNP** | **CHR** | **BP1** | **BP2** | **A1** | **A2** | **GP** | **GENE** | **MAF** | **HWE_P** | **BETA** | **P** |
| --- | --- | --- | --- | --- | --- | --- | --- | --- | --- | --- | --- | --- |
| DBP | rs11066280 | 12 | 112817783 | 112817783 | T | A | intronic | HECTD4 | 0.1732 | 0.7677 | -0.8991 | 7.70E-07 |
| DBP | rs11067763 | 12 | 116198341 | 116198341 | G | A | intergenic | TBX3(dist=1076372),MED13L(dist=198040) | 0.3322 | 0.5858 | -0.7157 | 9.27E-07 |
| FVC | rs10008568 | 4 | 89854192 | 89854192 | A | G | intronic | FAM13A | 0.4974 | 0.3582 | 1.492 | 2.35E-07 |
| FVC | rs2609261 | 4 | 89835485 | 89835485 | A | G | intronic | FAM13A | 0.4739 | 0.8271 | -1.464 | 3.37E-07 |
| FVC | rs2609260 | 4 | 89836819 | 89836819 | G | A | intronic | FAM13A | 0.4448 | 0.9023 | -1.471 | 3.56E-07 |
| FVC | rs2609264 | 4 | 89828080 | 89828080 | C | T | intronic | FAM13A | 0.4806 | 0.7898 | -1.434 | 6.00E-07 |
| FVC | rs1458551 | 4 | 89812242 | 89812242 | T | C | intronic | FAM13A | 0.4758 | 0.7896 | -1.419 | 8.32E-07 |
| log(GLU0) | rs7754840 | 6 | 20661250 | 20661250 | C | G | intronic | CDKAL1 | 0.478 | 1 | 0.01488 | 6.25E-09 |
| log(GLU0) | rs9460546 | 6 | 20663632 | 20663632 | G | T | intronic | CDKAL1 | 0.4824 | 0.7522 | 0.01449 | 1.25E-08 |
| log(GLU0) | rs10946398 | 6 | 20661034 | 20661034 | G | T | intronic | CDKAL1 | 0.4817 | 0.4826 | 0.01403 | 3.44E-08 |
| log(GLU0) | rs6456368 | 6 | 20659806 | 20659806 | C | T | intronic | CDKAL1 | 0.4814 | 0.08132 | 0.01404 | 3.27E-08 |
| log(GLU0) | rs9348440 | 6 | 20641336 | 20641336 | A | G | intronic | CDKAL1 | 0.4796 | 0.3449 | 0.01386 | 5.16E-08 |
| log(GLU0) | rs1799884 | 7 | 44229068 | 44229068 | A | G | upstream | GCK | 0.1872 | 0.9051 | 0.01889 | 5.62E-09 |
| log(GLU0) | rs10965245 | 9 | 22130515 | 22130515 | T | C | intergenic | CDKN2B-AS1(dist=9422),DMRTA1(dist=316325) | 0.3938 | 0.05408 | -0.01268 | 7.05E-07 |
| GLU120_log | rs4371338 | 2 | 5838030 | 5838030 | A | G | UTR3 | SOX11(NM_003108:c.*3851A>G) | 0.4852 | 0.6113 | 0.02584 | 2.08E-07 |
| GLU120_log | rs12229654 | 12 | 111414461 | 111414461 | G | T | intergenic | LINC01405(dist=39211),CUX2(dist=57367) | 0.1445 | 1 | -0.03548 | 5.00E-07 |
| GLU120_log | rs2074356 | 12 | 112645401 | 112645401 | T | C | intronic | HECTD4 | 0.1483 | 0.2317 | -0.03442 | 7.00E-07 |
| HB | rs5756505 | 22 | 37467354 | 37467354 | C | G | intronic | TMPRSS6 | 0.4979 | 0.9229 | 0.1125 | 2.61E-13 |
| HB | rs3768751 | 2 | 46346716 | 46346716 | G | A | intronic | PRKCE | 0.1796 | 1 | -0.113 | 1.79E-08 |
| HB | rs2218660 | 2 | 46347792 | 46347792 | C | A | intronic | PRKCE | 0.1802 | 0.5393 | -0.1128 | 1.99E-08 |
| HB | rs1005478 | 22 | 37478254 | 37478254 | A | G | intronic | TMPRSS6 | 0.4854 | 0.9807 | -0.07863 | 3.23E-07 |
| HB | rs17034571 | 2 | 46345932 | 46345932 | G | A | intronic | PRKCE | 0.1381 | 0.05676 | -0.1108 | 6.98E-07 |
| HB | rs17034573 | 2 | 46345957 | 46345957 | A | G | intronic | PRKCE | 0.1471 | 0.5311 | -0.1072 | 8.82E-07 |
| HB | rs4693156 | 4 | 88007227 | 88007227 | G | A | intronic | AFF1 | 0.4453 | 0.6596 | 0.07622 | 9.06E-07 |
| log(HBA1C) | rs7754840 | 6 | 20661250 | 20661250 | C | G | intronic | CDKAL1 | 0.478 | 1 | 0.01254 | 5.41E-11 |
| log(HBA1C) | rs9460546 | 6 | 20663632 | 20663632 | G | T | intronic | CDKAL1 | 0.4824 | 0.7522 | 0.01242 | 6.14E-11 |
| log(HBA1C) | rs10946398 | 6 | 20661034 | 20661034 | G | T | intronic | CDKAL1 | 0.4817 | 0.4826 | 0.0121 | 1.85E-10 |
| log(HBA1C) | rs6456368 | 6 | 20659806 | 20659806 | C | T | intronic | CDKAL1 | 0.4814 | 0.08132 | 0.0121 | 2.03E-10 |
| log(HBA1C) | rs9348440 | 6 | 20641336 | 20641336 | A | G | intronic | CDKAL1 | 0.4796 | 0.3449 | 0.01172 | 7.18E-10 |
| log(HBA1C) | rs7767391 | 6 | 20725240 | 20725240 | T | C | intronic | CDKAL1 | 0.474 | 0.6979 | -0.01097 | 7.04E-09 |
| log(HBA1C) | rs7747752 | 6 | 20725423 | 20725423 | C | G | intronic | CDKAL1 | 0.4744 | 0.5937 | -0.01088 | 9.44E-09 |
| log(HBA1C) | rs9465871 | 6 | 20717255 | 20717255 | T | C | intronic | CDKAL1 | 0.4505 | 0.8835 | -0.01053 | 2.57E-08 |
| log(HBA1C) | rs2328549 | 6 | 20718240 | 20718240 | A | T | intronic | CDKAL1 | 0.4363 | 0.768 | -0.0104 | 4.00E-08 |
| log(HBA1C) | rs2328529 | 6 | 20631953 | 20631953 | A | C | intronic | CDKAL1 | 0.4227 | 0.8236 | 0.01036 | 5.83E-08 |
| log(HDL) | rs10503669 | 8 | 19847690 | 19847690 | T | G | intergenic | LPL(dist=22920),SLC18A1(dist=154676) | 0.1207 | 0.003444 | 0.04214 | 7.99E-18 |
| log(HDL) | rs17482753 | 8 | 19832646 | 19832646 | T | G | intergenic | LPL(dist=7876),SLC18A1(dist=169720) | 0.1241 | 0.1333 | 0.04232 | 3.20E-18 |
| log(HDL) | rs17410962 | 8 | 19848080 | 19848080 | A | G | intergenic | LPL(dist=23310),SLC18A1(dist=154286) | 0.1242 | 0.08469 | 0.042 | 5.31E-18 |
| log(HDL) | rs16940212 | 15 | 58694020 | 58694020 | T | G | intergenic | ALDH1A2 | 0.3414 | 0.9786 | 0.02988 | 2.08E-18 |
| log(HDL) | rs16940174 | 15 | 58686338 | 58686338 | A | G | intergenic | ALDH1A2 | 0.3294 | 0.2286 | 0.02933 | 9.92E-18 |
| log(HDL) | rs261301 | 15 | 58686939 | 58686939 | A | G | intergenic | ALDH1A2 | 0.3262 | 0.869 | 0.02926 | 2.62E-17 |
| log(HDL) | rs16940170 | 15 | 58684282 | 58684282 | A | G | intergenic | ALDH1A2 | 0.3262 | 0.68 | 0.02911 | 4.17E-17 |
| log(HDL) | rs495348 | 15 | 58687790 | 58687790 | G | C | intergenic | ALDH1A2 | 0.3253 | 0.7405 | 0.02828 | 2.97E-16 |
| log(HDL) | rs4922117 | 8 | 19852586 | 19852586 | G | A | intergenic | LPL(dist=27816),SLC18A1(dist=149780) | 0.2077 | 0.418 | 0.0316 | 2.13E-15 |
| log(HDL) | rs17411031 | 8 | 19852310 | 19852310 | G | C | intergenic | LPL(dist=27540),SLC18A1(dist=150056) | 0.2092 | 0.6877 | 0.03125 | 3.57E-15 |
| HEIGHT | rs10513137 | 3 | 141143430 | 141143430 | A | G | intronic | ZBTB38 | 0.2585 | 0.2971 | 0.5194 | 5.20E-07 |
| HEIGHT | rs17038182 | 1 | 118868405 | 118868405 | G | C | intergenic | SPAG17(dist=140557),TBX15(dist=557261) | 0.4187 | 0.315 | -0.4648 | 7.12E-07 |
| LDL | rs599839 | 1 | 109822166 | 109822166 | G | A | downstream | PSRC1 | 0.06456 | 0.1925 | -5.886 | 1.53E-11 |
| LDL | rs12654264 | 5 | 74648603 | 74648603 | T | A | intronic | HMGCR | 0.4758 | 0.8085 | -2.625 | 1.41E-09 |
| LDL | rs3846663 | 5 | 74655726 | 74655726 | C | T | intronic | HMGCR | 0.4749 | 0.7712 | -2.593 | 2.29E-09 |
| LDL | rs2335418 | 5 | 74603479 | 74603479 | G | A | intergenic | ANKRD31(dist=70776),HMGCR(dist=29514) | 0.4232 | 0.5689 | -2.6 | 3.20E-09 |
| LDL | rs4045166 | 5 | 74909446 | 74909446 | G | C | intronic | ANKDD1B | 0.3326 | 0.3137 | 2.727 | 3.55E-09 |
| LDL | rs6861279 | 5 | 74919409 | 74919409 | T | C | intronic | ANKDD1B | 0.3386 | 0.1772 | 2.718 | 3.67E-09 |
| LDL | rs10942739 | 5 | 74786083 | 74786083 | T | C | intronic | COL4A3BP | 0.3325 | 0.276 | 2.709 | 4.61E-09 |
| LDL | rs7703051 | 5 | 74625487 | 74625487 | G | T | intergenic | ANKRD31(dist=92784),HMGCR(dist=7506) | 0.4705 | 0.7896 | -2.5 | 8.36E-09 |
| LDL | rs34340 | 5 | 74932099 | 74932099 | C | A | intronic | ANKDD1B | 0.3394 | 0.2463 | 2.509 | 4.94E-08 |
| LDL | rs688 | 19 | 11227602 | 11227602 | T | C | exonic | LDLR | 0.136 | 0.797 | 3.402 | 6.63E-08 |
| TCHL | rs599839 | 1 | 109822166 | 109822166 | G | A | downstream | PSRC1 | 0.06456 | 0.1925 | -6.822 | 1.19E-12 |
| TCHL | rs6861279 | 5 | 74919409 | 74919409 | T | C | intronic | ANKDD1B | 0.3386 | 0.1772 | 2.784 | 3.99E-08 |
| TCHL | rs780092 | 2 | 27743154 | 27743154 | G | A | intronic | GCKR | 0.3248 | 0.2948 | -3.365 | 4.63E-11 |
| TCHL | rs17321515 | 8 | 126486409 | 126486409 | T | C | intergenic | TRIB1(dist=35762),LINC00861(dist=448358) | 0.4425 | 0.04178 | 2.782 | 4.20E-09 |
| TCHL | rs2980875 | 8 | 126481747 | 126481747 | A | G | intergenic | TRIB1(dist=31100),LINC00861(dist=453020) | 0.4395 | 0.001112 | 2.672 | 1.62E-08 |
| TCHL | rs1881396 | 2 | 27844601 | 27844601 | G | T | UTR3 | ZNF512 | 0.3313 | 0.8699 | -2.793 | 3.18E-08 |
| TCHL | rs2980867 | 8 | 126487691 | 126487691 | A | C | intergenic | TRIB1(dist=37044),LINC00861(dist=447076) | 0.3136 | 0.0493 | 2.79 | 3.72E-08 |
| TCHL | rs2980862 | 8 | 126484638 | 126484638 | G | C | intergenic | TRIB1(dist=33991),LINC00861(dist=450129) | 0.3135 | 0.04033 | 2.766 | 4.82E-08 |
| TCHL | rs2954018 | 8 | 126477153 | 126477153 | C | T | intergenic | TRIB1(dist=26506),LINC00861(dist=457614) | 0.3133 | 0.02288 | 2.755 | 5.28E-08 |
| TCHL | rs6734059 | 2 | 27808154 | 27808154 | C | T | intronic | ZNF512 | 0.3357 | 0.8921 | -2.724 | 6.36E-08 |
| log(TG) | rs6589566 | 11 | 116652423 | 116652423 | C | T | intronic | ZPR1 | 0.2169 | 0.3545 | 0.1113 | 7.90E-38 |
| log(TG) | rs180349 | 11 | 116611827 | 116611827 | A | T | intergenic | LINC00900(dist=980909),BUD13(dist=7059) | 0.2265 | 0.752 | 0.1065 | 8.86E-35 |
| log(TG) | rs603446 | 11 | 116654435 | 116654435 | T | C | intronic | ZPR1 | 0.2252 | 0.3676 | -0.08646 | 2.02E-24 |
| log(TG) | rs11216126 | 11 | 116617240 | 116617240 | C | A | intergenic | LINC00900(dist=986322),BUD13(dist=1646) | 0.201 | 0.4981 | -0.07406 | 1.06E-16 |
| log(TG) | rs10503669 | 8 | 19847690 | 19847690 | T | G | intergenic | LPL(dist=22920),SLC18A1(dist=154676) | 0.1207 | 0.003444 | -0.08902 | 1.63E-16 |
| log(TG) | rs17482753 | 8 | 19832646 | 19832646 | T | G | intergenic | LPL(dist=7876),SLC18A1(dist=169720) | 0.1241 | 0.1333 | -0.0871 | 4.09E-16 |
| log(TG) | rs17410962 | 8 | 19848080 | 19848080 | A | G | intergenic | LPL(dist=23310),SLC18A1(dist=154286) | 0.1242 | 0.08469 | -0.08679 | 4.85E-16 |
| log(TG) | rs780094 | 2 | 27741237 | 27741237 | C | T | intronic | GCKR | 0.4626 | 0.9806 | -0.05656 | 2.60E-15 |
| log(TG) | rs7115242 | 11 | 116908283 | 116908283 | T | C | intronic | SIK3 | 0.2796 | 0.133 | 0.05987 | 8.66E-14 |
| log(TG) | rs4244457 | 8 | 19899046 | 19899046 | T | C | intergenic | LPL(dist=74276),SLC18A1(dist=103320) | 0.3261 | 0.3352 | -0.05611 | 2.37E-13 |
| WAIST | rs4121165 | 1 | 78276977 | 78276977 | T | C | intronic | FAM73A | 0.4491 | 0.714 | -0.6915 | 8.70E-07 |
| WAIST | rs11000212 | 10 | 73955652 | 73955652 | G | C | intronic | ASCC1 | 0.2044 | 0.6555 | 0.8562 | 8.90E-07 |
| WEIGHT | rs9739560 | 12 | 118461574 | 118461574 | A | T | intronic | RFC5 | 0.2551 | 0.4644 | 0.8238 | 8.18E-07 |

Table S2. Result of GWAS with $\boldsymbol{B}_{\mathbf{1}}$as response.

| **Trait** | **SNP** | **CHR** | **BP1** | **BP2** | **A1** | **A2** | **GP** | **GENE** | **MAF** | **HWE_P** | **BETA** | **P** |
| --- | --- | --- | --- | --- | --- | --- | --- | --- | --- | --- | --- | --- |
| BMI | rs17771607 | 9 | 24260531 | 24260531 | G | C | intergenic | ELAVL2(dist=434468),IZUMO3(dist=282682) | 0.2172 | 0.2266 | -0.01692 | 7.98E-06 |
| BMI | rs17698894 | 9 | 24241833 | 24241833 | A | G | intergenic | ELAVL2(dist=415770),IZUMO3(dist=301380) | 0.2245 | 0.252 | -0.01691 | 6.33E-06 |
| BMI | rs1329364 | 9 | 24240162 | 24240162 | C | A | intergenic | ELAVL2(dist=414099),IZUMO3(dist=303051) | 0.2248 | 0.282 | -0.0169 | 6.47E-06 |
| BMI | rs41505847 | 9 | 24240987 | 24240987 | T | C | intergenic | ELAVL2(dist=414924),IZUMO3(dist=302226) | 0.2247 | 0.2246 | -0.01667 | 8.42E-06 |
| BMI | rs13015992 | 2 | 18256919 | 18256919 | G | T | intergenic | KCNS3(dist=142694),RDH14(dist=479070) | 0.3735 | 0.4228 | 0.01489 | 4.22E-06 |
| BMI | rs1485315 | 8 | 120071898 | 120071898 | C | T | intergenic | TNFRSF11B(dist=107515),COLEC10(dist=7526) | 0.354 | 0.9368 | -0.01508 | 4.56E-06 |
| BMI | rs6897876 | 5 | 141687639 | 141687639 | C | T | intergenic | NDFIP1(dist=153631),SPRY4(dist=2353) | 0.2682 | 0.6441 | 0.01607 | 6.45E-06 |
| BMI | rs6580240 | 5 | 141684389 | 141684389 | T | A | intergenic | NDFIP1(dist=150381),SPRY4(dist=5603) | 0.2739 | 0.01064 | 0.01554 | 7.82E-06 |
| BMI | rs1536856 | 9 | 24238655 | 24238655 | A | C | intergenic | ELAVL2(dist=412592),IZUMO3(dist=304558) | 0.2244 | 0.2818 | -0.01656 | 9.90E-06 |
| DBP | rs10759930 | 9 | 120461621 | 120461621 | G | A | intergenic | LOC101928797(dist=42317),TLR4(dist=4832) | 0.395 | 0.7022 | -0.1034 | 9.53E-06 |
| FEV1 | rs2272402 | 3 | 11075461 | 11075461 | A | G | intronic | SLC6A1 | 0.07363 | 0.1473 | -0.5823 | 1.22E-08 |
| FEV1 | rs7209788 | 17 | 80415914 | 80415914 | T | C | upstream | NARF | 0.07118 | 0.522 | -0.5469 | 3.36E-07 |
| FEV1 | rs4789777 | 17 | 80394530 | 80394530 | G | T | intronic | HEXDC | 0.07508 | 0.6621 | -0.4787 | 4.60E-06 |
| FEV1 | rs4740238 | 9 | 134308491 | 134308491 | A | G | intronic | PRRC2B | 0.24 | 0.1043 | 0.2966 | 6.46E-06 |
| FEV1\FVC | rs450719 | 1 | 83163893 | 83163893 | A | G | intergenic | ADGRL2(dist=705471),LINC01361(dist=275673) | 0.206 | 0.1497 | 0.01449 | 9.78E-06 |
| FVC | rs2272402 | 3 | 11075461 | 11075461 | A | G | intronic | SLC6A1 | 0.07363 | 0.1473 | -0.595 | 1.40E-09 |
| FVC | rs17751180 | 2 | 77661105 | 77661105 | G | A | intronic | LRRTM4 | 0.2694 | 0.07987 | -0.2842 | 1.45E-06 |
| FVC | rs17586294 | 3 | 103378346 | 103378346 | A | G | intergenic | MIR548AB(dist=135386),MIR548A3(dist=525130) | 0.0547 | 0.000309 | -0.7694 | 1.73E-06 |
| FVC | rs7771273 | 6 | 85948871 | 85948871 | A | G | intergenic | TBX18(dist=474917),NT5E(dist=210431) | 0.06211 | 0.2493 | -0.5234 | 1.90E-06 |
| FVC | rs11697918 | 20 | 895325 | 895325 | G | A | intronic | ANGPT4 | 0.3852 | 0.09069 | -0.2619 | 2.37E-06 |
| FVC | rs17206242 | 12 | 79402617 | 79402617 | T | C | intronic | SYT1 | 0.1712 | 1 | -0.3168 | 6.47E-06 |
| log(HBA1C) | rs10736703 | 11 | 60107747 | 60107747 | G | A | intronic | MS4A6E | 0.07957 | 0.2818 | -0.00173 | 1.33E-06 |
| HEIGHT | rs1156293 | 3 | 68781236 | 68781236 | G | A | UTR3 | FAM19A4(NM_182522:c.*1057C>T,  NM_001005527:c.*1057C>T) | 0.07132 | 0.1708 | -0.02157 | 1.22E-06 |
| HEIGHT | rs2797910 | 10 | 31297484 | 31297484 | G | T | intronic | ZNF438 | 0.4176 | 0.6013 | -0.01036 | 6.23E-06 |
| HEIGHT | rs1690629 | 10 | 31292078 | 31292078 | C | T | intronic | ZNF438 | 0.4167 | 0.5506 | -0.01035 | 6.38E-06 |
| HEIGHT | rs1771645 | 10 | 31314469 | 31314469 | T | G | intronic | ZNF438 | 0.416 | 0.5503 | -0.01022 | 8.23E-06 |
| HEIGHT | rs1690630 | 10 | 31291992 | 31291992 | T | C | intronic | ZNF438 | 0.4164 | 0.5506 | -0.01021 | 8.48E-06 |
| HEIGHT | rs2642190 | 10 | 31305623 | 31305623 | G | T | intronic | ZNF438 | 0.4161 | 0.5339 | -0.01017 | 9.32E-06 |
| SBP_log | rs1952425 | 14 | 100136363 | 100136363 | A | C | intronic | HHIPL1 | 0.1171 | 0.9069 | 0.00201 | 4.43E-06 |
| SBP_log | rs4706458 | 6 | 71382765 | 71382765 | G | A | intronic | SMAP1 | 0.08995 | 0.01182 | -0.00214 | 9.62E-06 |
| TCHL | rs12760791 | 1 | 211188937 | 211188937 | A | G | intronic | KCNH1 | 0.09884 | 0.1534 | -0.5491 | 8.80E-06 |
| log(TG) | rs1325523 | 1 | 216074957 | 216074957 | A | C | intronic | USH2A | 0.05049 | 0.8993 | -0.01007 | 5.60E-06 |
| WAIST | rs10899345 | 11 | 76747519 | 76747519 | G | A | intronic | B3GNT6 | 0.3721 | 0.8159 | 0.05745 | 2.70E-06 |
| WAIST | rs10899346 | 11 | 76747625 | 76747625 | A | T | intronic | B3GNT6 | 0.3721 | 0.6978 | 0.05726 | 2.85E-06 |
| WAIST | rs17048091 | 3 | 68925778 | 68925778 | G | A | intronic | FAM19A4 | 0.1427 | 0.000829 | 0.07844 | 2.94E-06 |
| WAIST | rs11237061 | 11 | 76748199 | 76748199 | T | C | intronic | B3GNT6 | 0.372 | 0.5692 | 0.05612 | 4.37E-06 |
| WAIST | rs10814123 | 9 | 34561808 | 34561808 | T | C | intronic | CNTFR | 0.363 | 0.7938 | -0.0552 | 7.60E-06 |
| WEIGHT | rs17771607 | 9 | 24260531 | 24260531 | G | C | intergenic | ELAVL2(dist=434468),IZUMO3(dist=282682) | 0.2172 | 0.2266 | -0.04419 | 5.82E-06 |
| WEIGHT | rs17698894 | 9 | 24241833 | 24241833 | A | G | intergenic | ELAVL2(dist=415770),IZUMO3(dist=301380) | 0.2245 | 0.252 | -0.04354 | 6.22E-06 |
| WEIGHT | rs1329364 | 9 | 24240162 | 24240162 | C | A | intergenic | ELAVL2(dist=414099),IZUMO3(dist=303051) | 0.2248 | 0.282 | -0.04339 | 6.76E-06 |
| WEIGHT | rs41505847 | 9 | 24240987 | 24240987 | T | C | intergenic | ELAVL2(dist=414924),IZUMO3(dist=302226) | 0.2247 | 0.2246 | -0.04291 | 8.29E-06 |
| WEIGHT | rs17197000 | 9 | 24244133 | 24244133 | T | A | intergenic | ELAVL2(dist=418070),IZUMO3(dist=299080) | 0.2185 | 0.4355 | -0.04322 | 9.57E-06 |
| WEIGHT | rs17256610 | 9 | 24246608 | 24246608 | T | A | intergenic | ELAVL2(dist=420545),IZUMO3(dist=296605) | 0.2176 | 0.2411 | -0.04311 | 9.80E-06 |


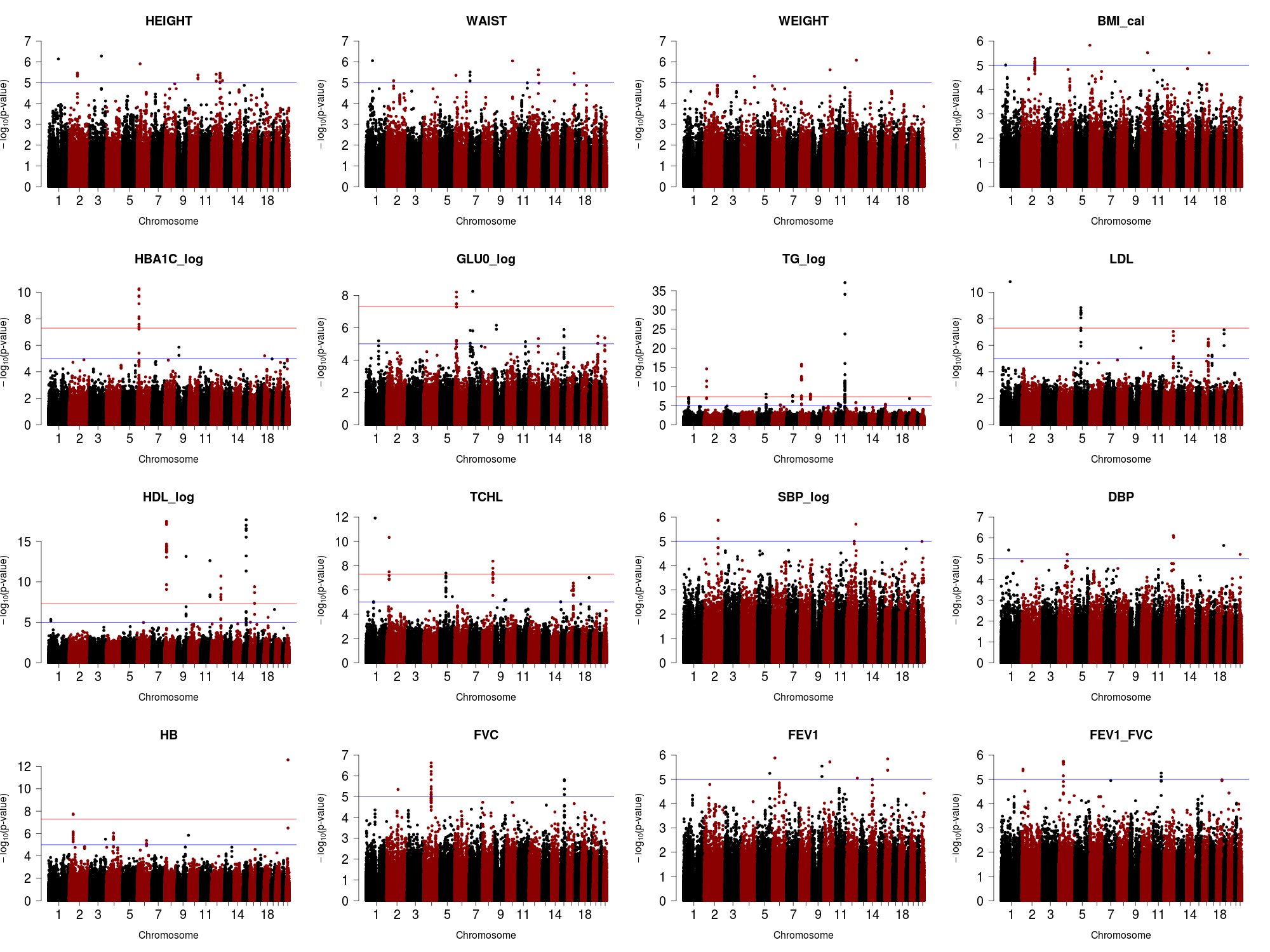


Figure S1. Manhattan Plot with $\boldsymbol{B}_{\mathbf{0}}$as response.


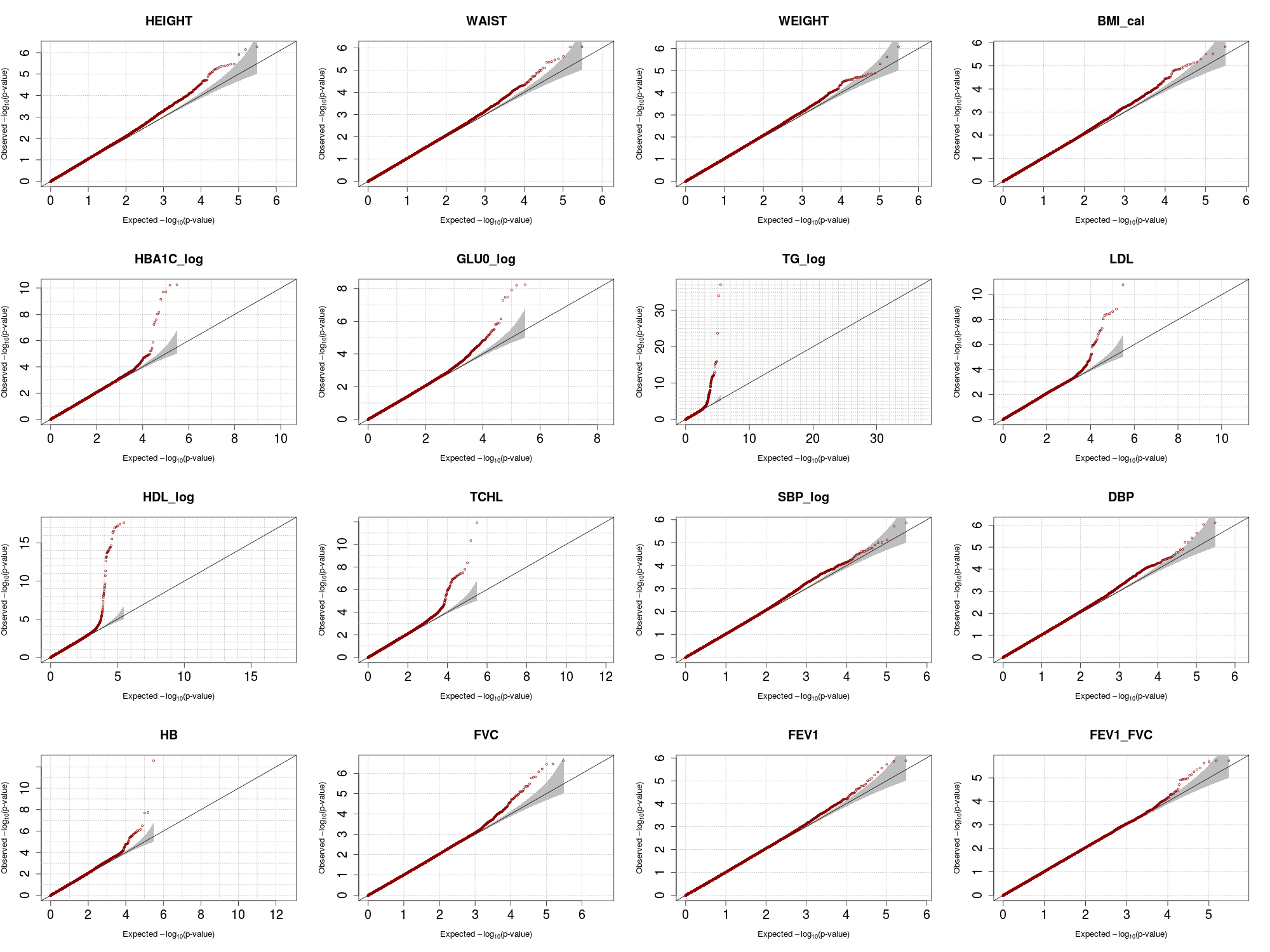


Figure S2. QQ plot with $\boldsymbol{B}_{\mathbf{0}}$as response.


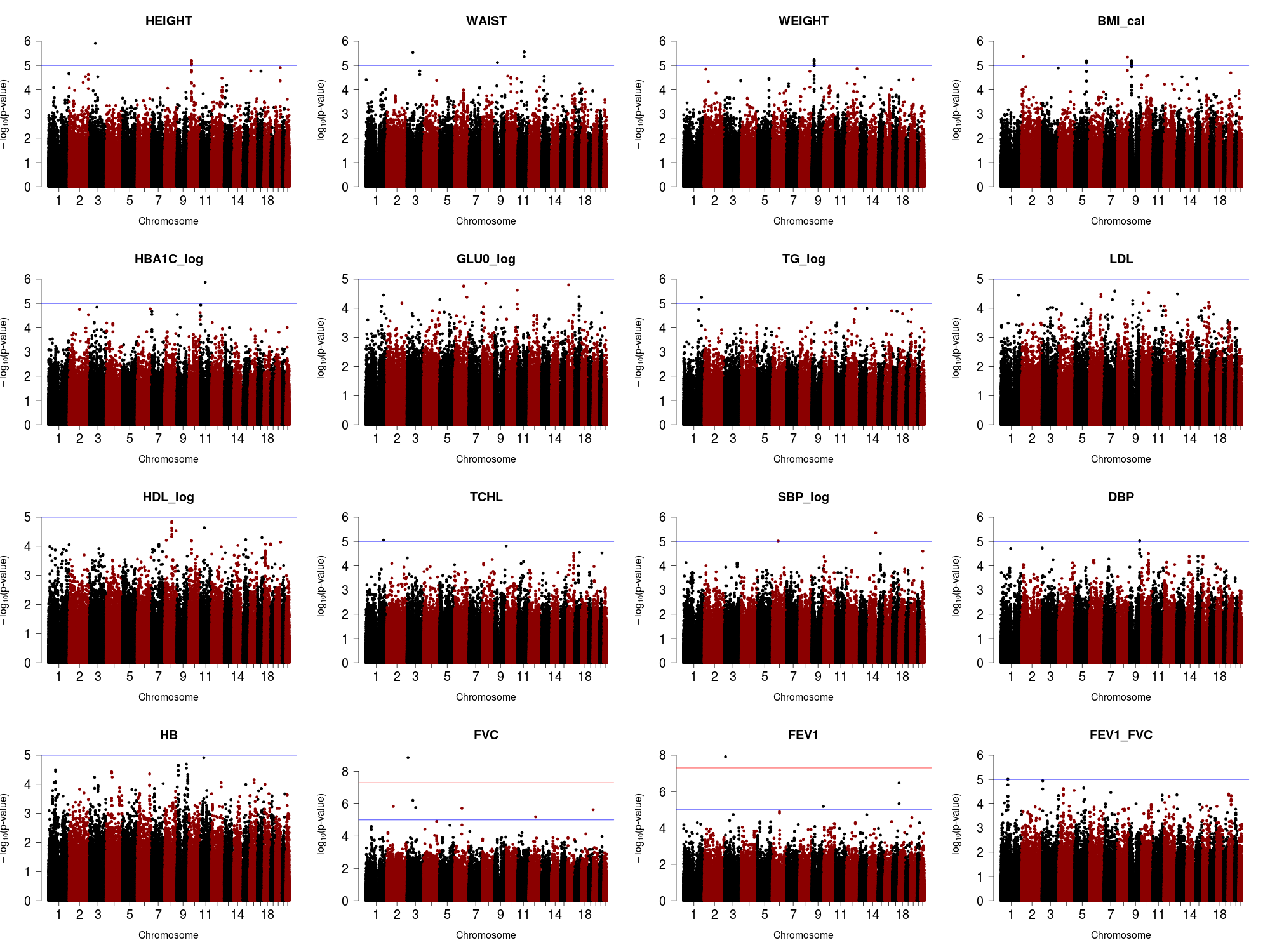


Figure S3. Manhattan plot with $\boldsymbol{B}_{\mathbf{1}}$ as response.


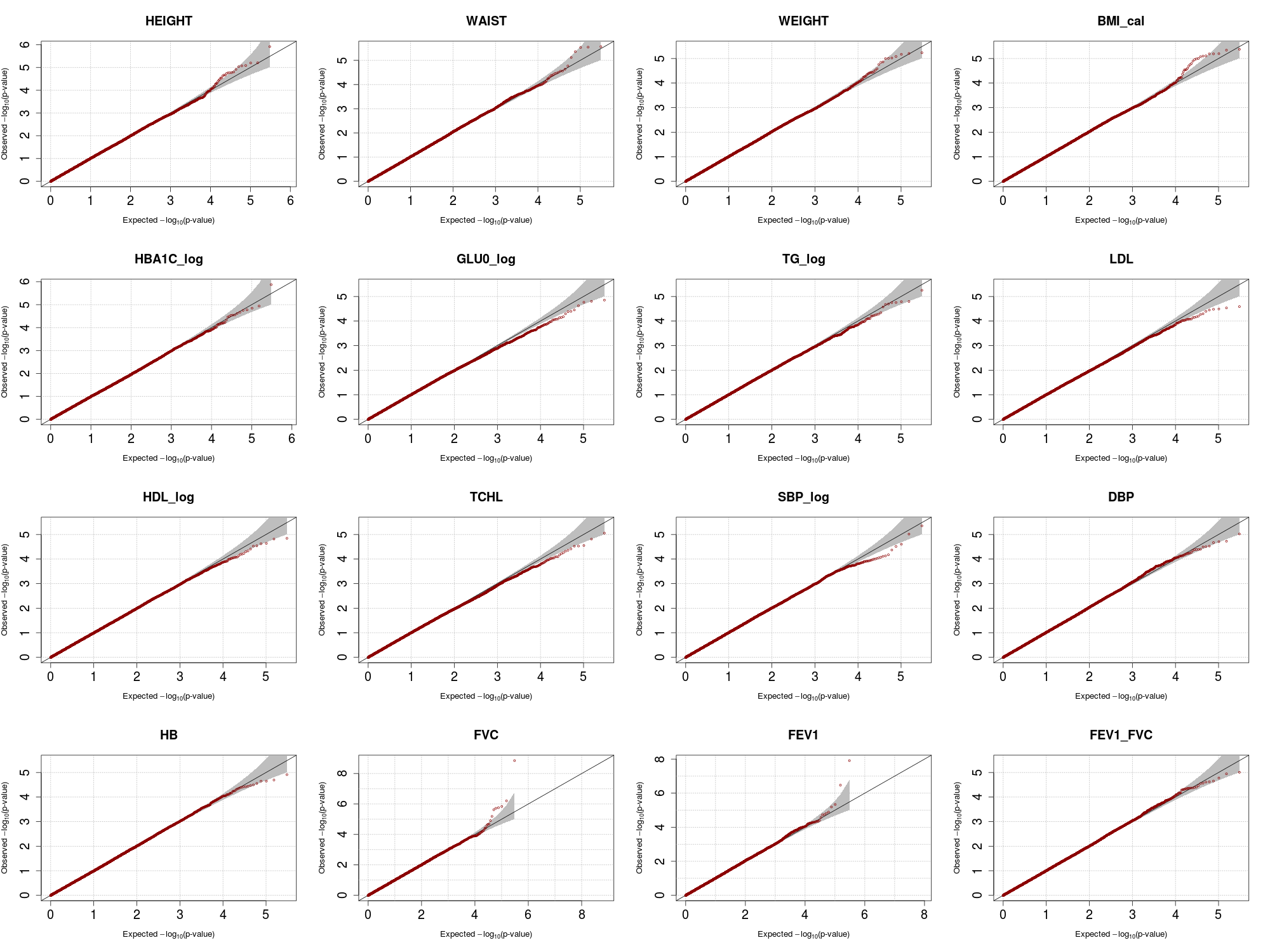


Figure S4. QQ plot with $\boldsymbol{B}_{\mathbf{1}}$ as response.


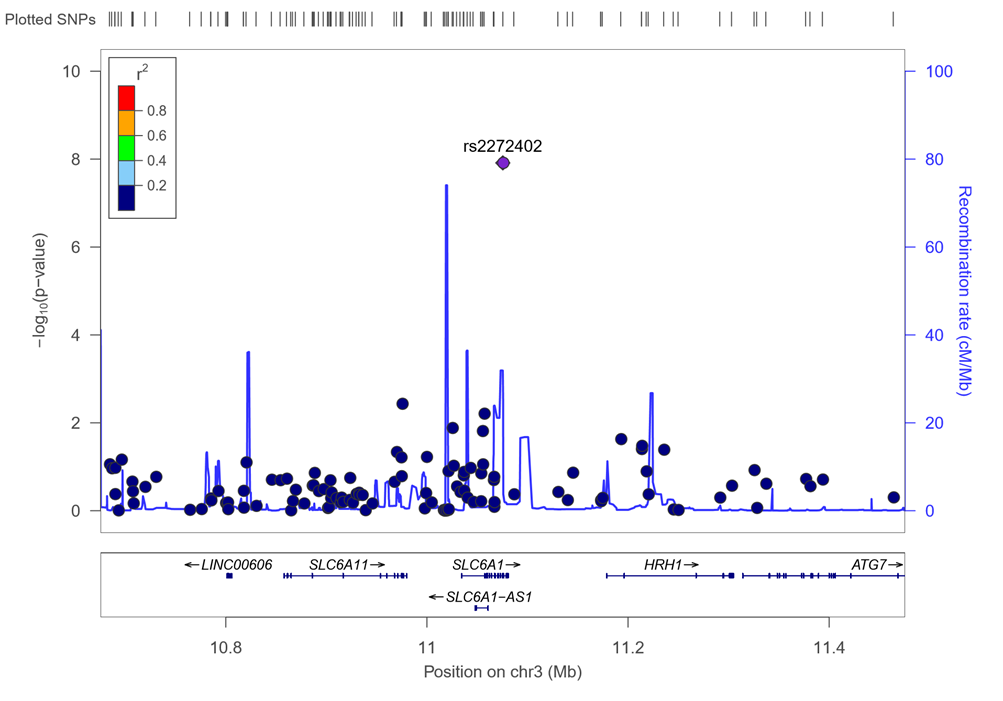


Figure S5. Regional plot of rs2272402 in FEV1 trait.


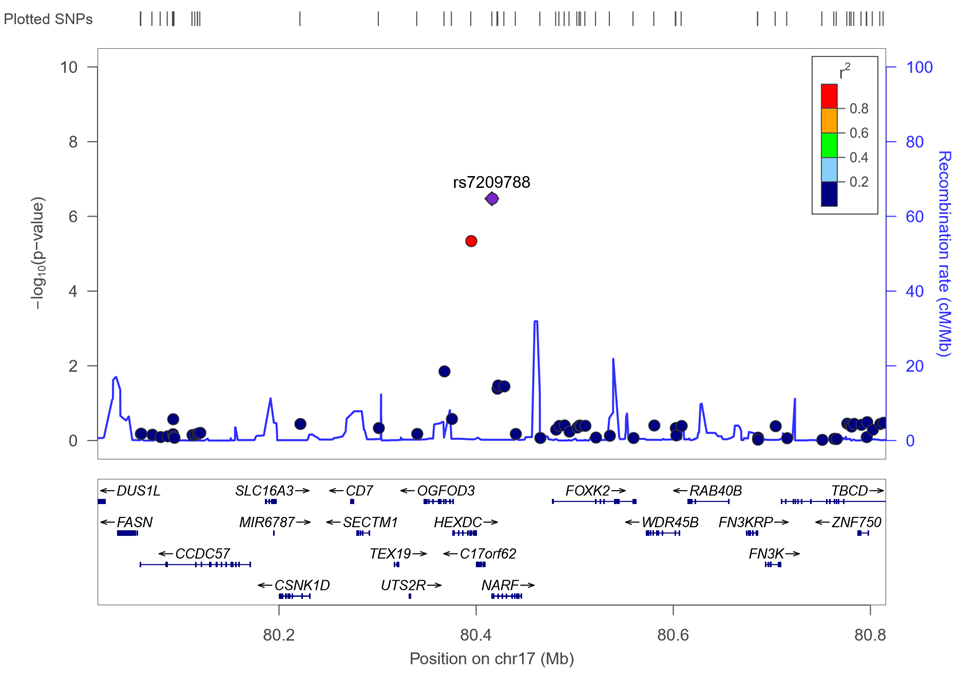


Figure S6. Regional Plot of rs7209788 in FEV1 trait.


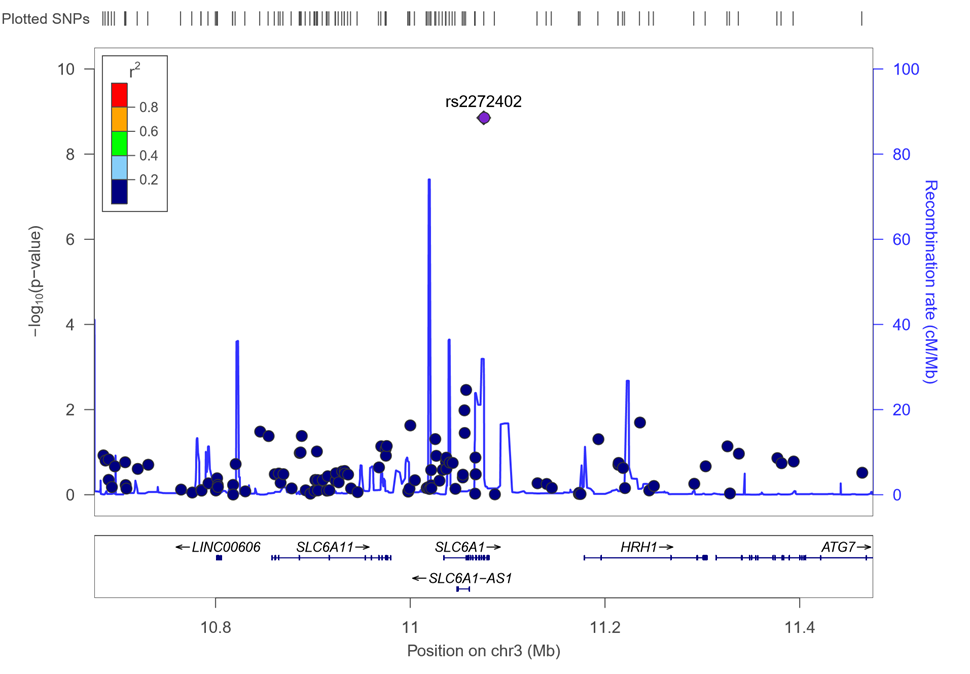


Figure S7. Regional plot of rs2272402 in FVC trait.
